# Burden of undiagnosed depression among older adults in India: a population based study

**DOI:** 10.1186/s12888-024-05684-5

**Published:** 2024-04-30

**Authors:** NB. Devikrishna, Nand Lal Mishra, Abhishek Singh

**Affiliations:** 1 International Institute for Population Sciences, 400088 Mumbai, India; 2 Department of Public Health & Mortality Studies, International Institute for Population Sciences, 400088 Mumbai, India

**Keywords:** Mental health, Depression, Geriatric mental health, Population ageing, Older adults

## Abstract

**Background:**

The study explored the levels and associated factors of undiagnosed depression among community-dwelling older Indian adults. It also identified the socio-demographic predictors of undiagnosed depression among the study population at national and state levels.

**Methods:**

The study employed data from the Longitudinal Ageing Study in India wave-I, 2017-18. Based on the data on depression from interviewee’s self-reporting and measurement on Composite International Diagnostic Interview– Short Form (CIDI-SF) and Centre for Epidemiological Studies- Depression scale (CES-D) scales, we estimated undiagnosed depression among older adults (age 60+). We estimated multivariable binary logistic regressions to examine the socio-demographic and health-related predictors of undiagnosed depression among older adults.

**Findings:**

8% (95% CI: 7.8–8.4) of the total older adults had undiagnosed depression on CIDI-SF scale and 5% (95% CI: 4.8–5.3) on the combined CIDI-SF and CES-D. Undiagnosed depression was higher among those who were widowed, worked in the past and currently not working, scheduled castes, higher educated and the richest. Lack of health insurance coverage, presence of any other physical or mental impairment, family history of Alzheimer’s/Parkinson’s disease/ psychotic disorder, lower self-rated health and poor life satisfaction were significant predictors of undiagnosed depression on both CIDI-SF and combined scales.

**Conclusion:**

To improve the health of older adults in India, targeted policy efforts integrating mental health screening, awareness campaigns and decentralization of mental healthcare to primary level is needed. Further research could explore the causal factors behind different levels of undiagnosed depression.

**Supplementary Information:**

The online version contains supplementary material available at 10.1186/s12888-024-05684-5.

## Background

The improvement in life expectancy has prepared all countries in the world to expect a considerable share of population to cross the age of 60, by 2030, every sixth person in the world will be an older adult [1]. Mental and physical health difficulties such as dementia, Alzheimer’s, functional disability etc. comes as the part and package of the phenomenon of population ageing. However, the growing tendency to associate depression with the process of biological aging is inherently flawed [2] especially when the single largest contribution to global disability and to suicides is attributable to it [3]. A World Health Organization (WHO) survey conducted in more than 180 countries shows that around 40% of them do not have a comprehensive mental health policy and about one-third of them lack specific budget allocation for the same [4]. In the context of growing older adult population and burden of mental health issues, institutional efforts for the promotion of healthy aging among older adults is crucial for the country.

India is graying as the share of older adults (60+) population has increased from 7.7% of the total at the dawn of millennium to 9.4% in 2017, and is expected to reach 19% (324 million) by 2050 [5]. Naturally, India is expected to witness rise in physical and mental health problems. A meta-analysis of the prevalence of depression among older adults in India during 1997–2016 found that one in every third older adult age 60 + was depressed, posing public health challenge for the country [6]. However, to address the huge burden of depression and the taboos associated with it, the country does not seem to have enough ministerial level policies and programs focusing especially on the mental health of older adults. India had introduced National Policy for Older Persons (NPOP) in 1999 to ensure financial support, food security, shelter and other facilities by state governments. The policy envisaged to make health, social and economic provision for older adults and to provide them protection from all sorts of abuses and exploitation [7]. Both NPOP and National Policy for Senior Citizens (2011) however give scant attention to mental health of older adults [8]. However, the onus cannot be fully transferred to the policy makers as the country had been looking at a big lacuna created by the lack of data and literature, until the release of Longitudinal Ageing Study in India (LASI) Wave-I data. LASI provides in-depth data on the cognitive aspects of Indian older adults using international scales and standards. On an average 8.3% of Indian older adults are measured for major probable depression on the Composite International Diagnostic Interview– Short Form (CIDI-SF) scale and 0.8% have reported diagnosed for depression [9]. The social change in the country that followed the phenomena of globalization, urbanization and disintegration of a well-knit joint family system has pushed the older adults into more social and economic vulnerabilities that are expected to take a toll on their mental health. Hence, an exhaustive understanding of depression among older adults by focusing on the factors that aggravates the cognitive conditions and those which can improve the same is needed at the moment.

One should not underestimate the importance of depression as a public health concern owing to its commonness, as around 5% of adults and 5.7% of older adults around the world are estimated to be suffering from it [10]. Projections by Global Burden of Disease Study shows depression as the leading cause of Disability Adjusted Life Years (DALY) in developing countries by 2020 [11]. Apart from being the leading cause of disability, depression is also one of the major reasons for deaths by suicide, even in India [12]. Studies have found that irrespective of the fact that the treatment for depression is available, 75% of people from lower-middle income countries did not receive any treatment due to reasons such as inaccessibility to the services, inadequate resources and personals and stigma associated with both mental health issues and their care-seeking [13]. Though the Indian academia has begun to look into the risk factors and other determinants of geriatric depression, the screening and diagnosis aspect has received only deficit attention.

The current study aims at understanding the level of undiagnosed depression among Indian older adults (60+) and their predictors using the LASI Wave-1 dataset. The study has utilised two international scales of measurement (i) Composite International Diagnostic Interview– Short Form (CIDI-SF) and (ii) Centre for Epidemiological Studies- Depression scale (CES-D) to measure depression among study population. CIDI-SF is a depression measurement scale that assesses major probable depression and other disorders such as generalized anxiety and Obsessive-Compulsive Disorder (OCDs) over the course of the past twelve months [14–16].. CES-D scale, on the other hand, measures depressive symptomatology rather than the illness severity [17–19]. Established literature has cautioned against the use of CES-D independently and suggests the application of it in combination with other well-established scales, owning to the concerns of reliability [20–23]. This study will hence utilise the CIDI-SF scale of measurement for estimating the depressive cases. In addition, the combined scale of measurement, that is CIDI-SF and CES-D together is also used in the current analysis to understand the older adults found depressed on both the scales. LASI Wave-1 has utilized the.

For more details on.

## Methods

### Data source

**Fig. 1:**
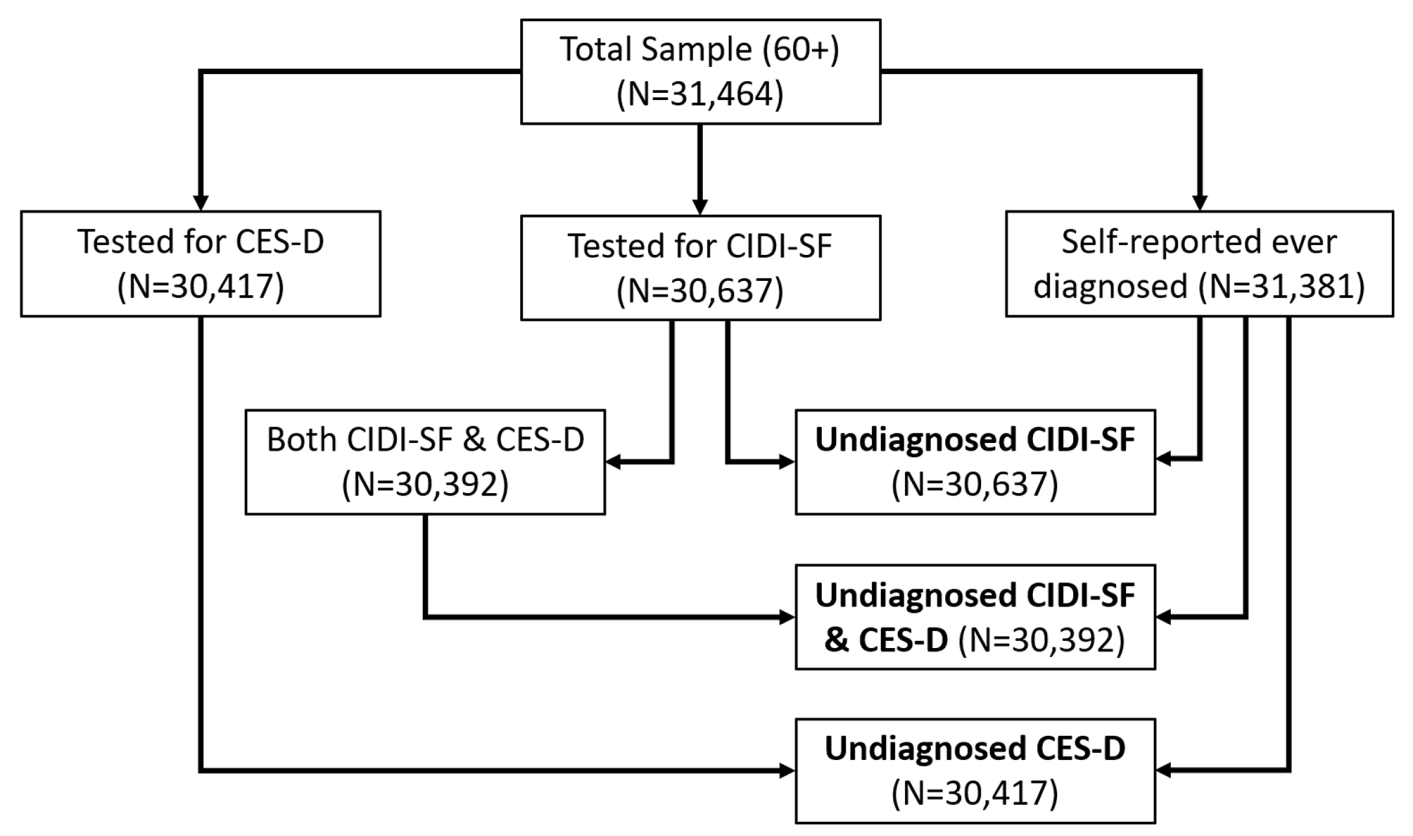
A: Sample size flow chart of undiagnosed depression among older adults age 60 and above, India (2017-18)

This study has primarily utilized the Longitudinal Aging Study in India (LASI) Wave-1 data, which analyses the health, demographic, and socio-economic nuances of the phenomenon of population ageing systematically. The survey was carried out in 35 states and union territories of the country (excluding Sikkim) and has a sample size of 72,250 adults aged 45 years and above and their spouses, irrespective of their age. The sampling procedure of the LASI Wave-1 included only the conventional unit of the household population and excluded the individuals living in other arrangements such as caring facilities, hostels, prisons, etc. LASI Wave-1 adopted a multi-stage stratified area-probability cluster sampling design with sub-districts/tehsils as primary sampling units (PSU) and village/urban wards as secondary sampling units (SSU)where a three-stage sampling design in rural areas and a four-stage sampling design in urban areas, within each state, was adopted. The list of sub-districts (Tehsils/Talukas) taken from the Census of 2011 was the sampling frame for the first stage from which the primary sampling units (PSU) are selected. Subsequently, villages (rural) and wards (urban) are chosen from these selected PSUs in the second sampling stage. The third stage in the rural areas involved the selection of households from these chosen villages. In contrast, the sampling in urban areas adopted a further stage of first randomly identifying one Census Enumeration Block (CEB) from each area and then identifying the households from the selected CEBs as the fourth step. Detailed information about sampling design, fieldwork, data collection tools and processing etc. are available elsewhere [24, 25]. The current study analysed information from older adults age 60 and above. Out of the 31,464 samples, a total of 30,637 and 30,392 respondents were included in the statistical analysis for undiagnosed depression on the CIDI-SF and combined scales, respectively. Respondents with missing diagnosis status information (83) or those who did not participate in the CIDI-SF (827) and CES-D (1047) testing were excluded from the analysis (See Fig.  1A).

The study did not require any ethical approval as publicly available secondary data source from a longitudinal study was utilised. LASI wave-1 has adhered to all the standard procedures and approved protocols, including informed consent to collect data from the participants.

### Variable description

#### Outcome variable

Undiagnosed depression defined on two scales (CIDI-SF and Combined CIDI-SF & CES-D) are the outcome variables of this study. In the LASI study, the Short Form Composite International Diagnostic Interview (CIDI-SF) is employed to estimate the prevalence of major depression based on diagnostic symptoms. This scale is designed to identify potential cases of major depression and has been validated for use in real-world settings. It is widely utilized in health surveys conducted among the general population (LASI, 2020). Total 7 symptoms-based questions for depression– Loss of interest, feeling tired, loss of appetite, trouble concentrating, feeling of worthlessness, thinking about death and trouble falling asleep were asked in LASI Wave-1. Scores on the CIDI-SF scale can range from 0 to 7, and a threshold of three or more is applied to determine the prevalence of probable major depression, following the methodology outlined by Kessler and Ustun [42].

The CES-D, short for the Center for Epidemiologic Studies Depression Scale, is a self-report questionnaire initially developed by Radloff in 1977. It serves as a screening tool for assessing depressive symptoms in the general population. The original CES-D scale consists of 20 items [43]. However, in the LASI study, a modified 10-item version with four response options was utilized. These 10 items comprise seven negative symptoms (including trouble concentrating, feeling depressed, low energy, fear, loneliness, being bothered by things, and experiencing everything as an effort) and three positive symptoms (feeling happy, hopeful, and satisfied). Respondents were asked to indicate the frequency of experiencing these symptoms over the week preceding the interview. Response categories included “rarely or never” (< 1 day), “sometimes” (1 or 2 days), “often” (3 or 4 days), and “most or all of the time” (5–7 days). (LASI, 2020). For the negative symptoms, a score of zero was assigned to “rarely or never” and “sometimes,” while a score of one was given to “often” and “most or all of the time.” Conversely, the scoring was reversed for positive symptoms. The overall score on this 10-item scale ranges from zero to 10, and a score of four or more is used as the threshold to calculate the prevalence of depressive symptoms (LASI, 2020).

**Fig. 2:**
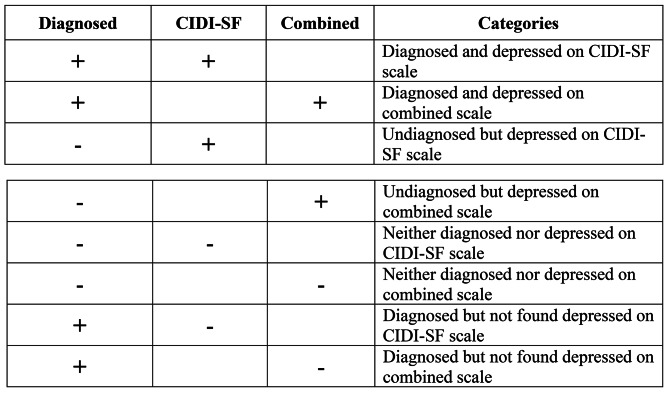
B: Defining undiagnosed depression in the study

In our study, older adults (60+) are defined as having depressive symptoms if they secure a score of four or more on a 0–10 score based CES-D scale. A score of three or more on a 0–7 CIDI-SF scale is defined as probable major depression among the older adults. Those individuals who fulfil both these criteria simultaneously are defined as the older adults who are measured depressed on both the scales of measurement. An older adult is defined as undiagnosed for depression on CIDI-SF or combined scale (CIDI-SF & CES-D) if he/she was measured as having depression on the respective scale and answered “no” to the question on self-reporting of ever diagnosed depression, where they have ever been told by a health professional as depressed. Figure 2B presents a matrix of self-reported diagnosis status and depression status on CIDI-SF and Combined scales. Outcome of our interest are, those who were– (1) Undiagnosed but depressed on CIDI-SF scale, and (2) Undiagnosed but depressed on combined scale.

#### Predictor variables

The study extracted data on certain background characteristics, such as urban-rural residence, sex (male, female), marital status (currently married, widowed, divorced/separated/deserted/others), living arrangement (living alone, living with spouse and/or others, living with spouse and children, living with children and others, and living with others), religion (Hindu, Muslim, Others), Caste/Tribe (Scheduled Castes, Scheduled Tribes, Other Backward Class, Others), educational attainment (no schooling, less than 5-years completed, 5–9 years completed, more than 10 years completed), work status (currently working, worked in the past but currently not working, never worked), monthly per capita expenditure (MPCE) quintile (poorest, poorer, middle, richer, richest), and geographic region of residence (North, Central, East, North-East, West and South India).

Based on prior studies, we included other predictor variables such as health insurance coverage, the presence of any diagnosed neurological or psychiatric issues (excluding depression), the existence of any physical disability, and a family history of Alzheimer’s, Parkinson’s disease, or psychotic disorders. These variables were categorized as “yes” or “no.” Additionally, we incorporated two summary measures: self-rated health (categorized as “good,” “moderate,” or “poor”) and life satisfaction (categorized as “low,” “medium,” or “high”) into our statistical analysis.

Self-rated health (SRH) (also known as self-assessed health or self-perceived health) refers to a single-item health measure in which individuals rate the current status of their health on a five-point scale from very good to very poor. The survey administered the question “overall, how is your health in general? Would you say it is very good, good, fair, poor or very poor?”, and coded these five responses into three categories, namely (i) good (very good, and good) (ii) moderate (fair), and (iii) poor (poor, and very poor).

Life satisfaction, a crucial measure of wellbeing, and successful aging of elderly is examined, and measured based on five statements, namely (i) in most ways, my life is close to ideal (ii) the conditions of my life are excellent (iii) I am satisfied with my life (iv) So far, I have got the important things I want in my life and (v) if I could live my life again, I would change almost nothing. The responses to the question were coded into seven categories of (i) strongly disagree (ii) somewhat disagree (iii) slightly disagree (iv) neither agree nor disagree (v) slightly agree (vi) somewhat agree, and (vii) strongly disagree. The scores on these categories are added up to construct a life-satisfaction scale on which a score of 5–20 indicates ‘low satisfaction’, 21–25 of ‘medium satisfaction’, and a ‘high satisfaction’ is indicated by scores ranging from 26 to 35.

### Data analysis

The study analysed the prevalence of undiagnosed depression among older adults with respect to multiple socio-economic, demographic, residence-related, and other predictors using cross-tabulation analysis. Bivariate associations were assessed by applying Chi Square test of independence. Two multivariable logistic regression models, one for the CIDI-SF and second for the combined scale, were estimated to delineate the factors associated with the outcome of interest. Before running the model, we ensured that there was no multicollinearity. We recorded pair-wise correlations between selected variables, and they all fell between − 0.3 and + 0.3, clearly indicating the absence of multicollinearity. Additionally, the Hosmer-Lemeshow test was applied to assess model fit. In both cases, insignificant p-values (> 0.05) were obtained from the test, indicating that the models are suitable for predicting the outcome variable. We carried out all analyses by utilising the survey weights given in LASI Wave 1 to ensure the nationally representative nature of the sample. All the analyses were done using STATA 15.0 (Stata Corp., College Station, USA) software.

## Results

### Summary statistics

**Fig. 3:**
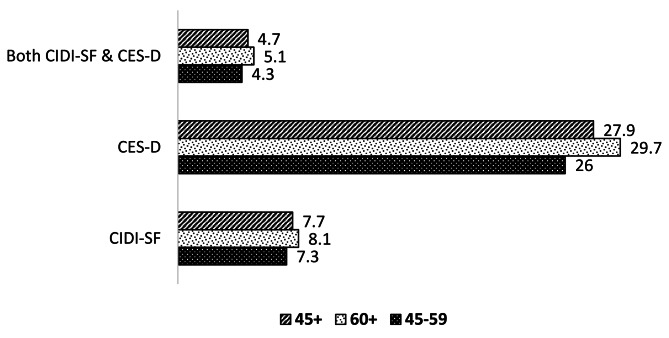
Undiagnosed depression among older adults, India, 2017-18

Figure 3 gives the summary estimates of the levels of undiagnosed depression among the total population for the age groups 45–59, 60+, and 45 + for CIDI-SF, CES-D, and combined scale. 8%, 30%, and 5% of older adults (60+) were not diagnosed for depression as per CIDI-SF, CES-D and combined scales respectively.

### Undiagnosed depression by socio-economic, demographic, residence-related, and other predictors

Table 1 provides population-weighted numbers and percentages of undiagnosed depression among older adults measured on CIDI-SF and combined scales by different predictors. The rates of undiagnosed depression were slightly higher among respondents who were from rural areas, were female, were widowed, lived alone, belonged to Muslim, belonged to Scheduled castes, had no schooling, had worked in the past but currently not working, belonged to the richest wealth quintile, and belonged to the Central part of India. These results were consistent on both CIDI-SF and combined scale. The rates of undiagnosed depression were also higher among those who did not have health insurance coverage, had any other diagnosed neurological/psychiatric issues except depression, suffered from physical/mental impairment, rated their health as poor on self-rated health, and had low life satisfaction. Test results indicates that all selected background characteristics are significantly associated with the undiagnosed status of older adults (60+) in India.

**Table 1:** Prevalence of undiagnosed depression among older adults on CIDI-SF and combined scales by selected background characteristics, India (2017-18)

Background characteristics	Undiagnosed CIDI-SF				Undiagnosed on combined scale			
	%	95% CI	Count	Chi2	%	95% CI	Count	Chi2
**Place of residence**				53.9***				28.4***
Rural	9	8.6–9.4	1,961		5.7	5.4-6.0	1,232	
Urban	5.7	5.3–6.1	508		3.5	3.2–3.9	311	
**Sex**				19.7***				31.7***
Male	7	6.6–7.4	1,016		4.1	3.8–4.4	591	
Female	9	8.5–9.4	1,453		5.9	5.6–6.3	952	
**Marital status**				64.1***				80.9***
Currently married	7.2	6.9–7.6	1,371		4.3	4.0-4.6	808	
Widowed	9.6	9.0-10.1	1,057		6.4	5.9–6.9	705	
Divorced/Separated/Deserted/Others	6.2	4.5–7.9	41		4.7	3.2–6.1	30	
**Living arrangement**				78.1***				101***
Living alone	12.8	11.2–14.5	226		8.8	7.3–10.1	152	
Living with spouse and/or others	7.9	7.2–8.6	480		4.7	4.2–5.3	284	
Living with spouse and children	6.9	6.5–7.4	880		4.1	3.8–4.5	518	
Living with children and others	8.3	7.7–8.9	704		5.5	5.0–6.0	462	
Living with others	10.8	9.3–12.3	180		7.8	6.5–9.1	126	
**Religion**				48.2***				52.9***
Hindu	8	7.7–8.4	2,031		5.2	4.9–5.4	1,296	
Muslim	8.9	8.0-9.8	293		5.3	4.5-6.0	172	
Others	7.2	6.4–7.9	144		3.7	3.2–4.3	74	
**Caste/tribe**				127.2***				77.5***
Scheduled caste	9.6	8.8–10.4	557		6.5	5.8–7.2	378	
Scheduled tribe	4.5	3.9–5.1	112		3	2.6–3.5	75	
Other backward classes	8.4	7.9–8.9	168		5.4	5.0-5.8	736	
None of the above	7.5	6.9-8.0	633		4.2	3.8–4.6	354	
**Education**				54.8***				55.9***
No schooling	8.9	8.5–9.4	1,549		5.9	5.5–6.2	1,005	
Less than 5 years completed	9.3	8.4–10.2	329		5.8	5.1–6.6	205	
5–9 years completed	6.9	6.2–7.5	376		3.8	3.3–4.3	208	
10 or more years completed	5	4.4–5.6	215		2.9	2.4–3.4	124	
**Work status**				21.1***				32.8***
Currently working	7.4	6.9–7.9	711		4.3	3.9–4.7	411	
Worked in past but currently not working	9.2	8.7–9.7	1,194		6.3	5.9–6.7	804	
Never worked	7	6.4–7.5	564		4.1	3.6–4.5	327	
**MPCE quintile**				19.5**				21.4***
Poorest	8.5	7.8–9.2	565		6.1	5.5–6.7	405	
Poorer	7.2	6.6–7.9	481		4	3.5–4.5	264	
Middle	7.6	6.9–8.2	480		4.7	4.2–5.3	299	
Richer	8	7.3–8.7	476		4.9	4.4–5.5	287	
Richest	9.3	8.5–10.0	467		5.8	5.2–6.4	288	
**Region**				322.7***				262.8***
North	6.6	5.9–7.2	257		3.7	3.2–4.2	144	
Central	13.8	12.7–14.8	883		9.3	8.4–10.2	592	
East	7.8	7.1–8.5	569		4.1	3.6–4.6	298	
North-East	5.1	4.4–5.8	47		2.4	1.9–2.9	22	
West	7.1	6.3-8.0	373		4.9	4.2–5.7	258	
South	5	4.5–5.4	340		3.4	3.0-3.8	228	
**Health insurance coverage**				27.7***				15.6***
No	8.6	8.2–8.9	2,147		5.4	5.1–5.7	1,346	
Yes	5.7	5.2–6.3	322		3.5	3.1-4.0	197	
**Any other diagnosed neurological/psychiatric issues except depression**				20.7***				33.5***
No	8	7.7–8.3	2,391		5	4.7–5.2	1,480	
Yes	10.8	8.4–13.1	79		8.7	6.5–10.8	63	
**Any physical/mental impairment**				151.7***				136.5***
No	7.4	7.1–7.7	2,068		4.6	4.3–4.8	1,274	
Yes	15.6	14.1–17.0	401		10.6	9.3–11.8	269	
**Family history of Alzheimer’s/Parkinson’s disease/Psychotic disorder**				60.6***				58.1***
No	7.8	7.5–8.1	2,259		4.8	4.6–5.1	1,388	
Yes	13.6	11.9–15.4	210		10.2	8.6–11.7	154	
**Self-rated health**				604.8***				518.4***
Good	4.1	3.7–4.5	386		2.2	1.9–2.5	202	
Moderate	6.9	6.5–7.3	956		4.2	3.8–4.5	571	
Poor	15.2	14.4–16.0	1,126		10.4	9.7–11.1	769	
**Life satisfaction**				298.2***				361.9***
Low	12.3	11.6–12.9	1,194		8.6	8.0-9.2	841	
Medium	7.3	6.7–7.9	517		4.5	4.0–5.0	308	
High	5.5	5.1–5.9	758		2.9	2.6–3.1	393	
**Total**	**8.1**	**7.8–8.4**	**2,469**		**5.1**	**4.8–5.3**	**1,543**	

***p < 0.001, **p < 0.01, *p < 0.05

Table 2 presents the rates of undiagnosed depression among different states and Union Territories (UTs) of the country. While Madhya Pradesh (14.4%) had the highest percentage of undiagnosed depression, Gujarat (3.3%) had the lowest. Undiagnosed depression was also high in Uttar Pradesh (13.1%) and Bihar (10.1%).

**Table 2:** Undiagnosed depression among older adults on CIDI-SF and combined scales by states and UTs, India, 2017-18

States & UTs	Undiagnosed CIDI-SF			Undiagnosed CIDI-SF & CES-D		
	%	95% CI	n	%	95% CI	n
Jammu & Kashmir	4.4	2.9–5.9	14	2.3	1.2–3.4	7
Himachal Pradesh	6.1	4.2-8.0	12	2.3	1.1–3.5	4
Punjab	9.0	7.2–10.8	71	3.6	2.4–4.8	28
Chandigarh	*	*	*	*	*	*
Uttarakhand	7.4	5.4–9.4	20	3.5	2.1–4.9	9
Haryana	5.3	3.8–6.8	30	3.6	2.3–4.8	20
Delhi	10.2	7.5–12.9	39	6.9	4.6–9.1	26
Rajasthan	5.6	4.2-7.0	97	3.6	2.5–4.8	62
Uttar Pradesh	13.7	12.3–15.2	642	8.9	7.7–10.2	415
Bihar	10.1	8.7–11.5	265	4.6	3.6–5.5	118
Arunachal Pradesh	*	*	*	*	*	*
Nagaland	*	*	*	*	*	*
Manipur	*	*	*	*	*	*
Mizoram	*	*	*	*	*	*
Tripura	*	*	*	*	*	*
Meghalaya	*	*	*	*	*	*
Assam	6.8	5.0-8.5	35	3.1	1.9–4.4	16
West Bengal	7.2	5.9–8.5	159	4.8	3.7–5.9	107
Jharkhand	6.5	5.1–7.9	51	3.5	2.4–4.6	27
Odisha	4.5	3.3–5.7	53	2.0	1.2–2.7	23
Chhattisgarh	3.4	2.1–4.6	17	2.3	1.2–3.4	12
Madhya Pradesh	16.1	14.1–18.2	282	11.6	9.8–13.4	198
Gujarat	3.1	2.0-4.2	41	1.6	0.8–2.4	21
Daman & Diu	*	*	*	*	*	*
Dadra & Nagar Haveli	*	*	*	*	*	*
Maharashtra	8.8	7.4–10.1	287	6.3	5.2–7.5	205
Andhra Pradesh	3.7	2.5–4.8	51	2.1	1.2–2.9	29
Karnataka	6.5	4.9-8.0	82	4.9	3.5–6.2	60
Goa	*	*	*	*	*	*
Lakshadweep	*	*	*	*	*	*
Kerala	5.5	4.2–6.8	66	3.5	2.4–4.5	41
Tamil Nadu	5.0	3.9–6.1	117	3.9	2.9–4.9	91
Puducherry	*	*	*	*	*	*
Andaman & Nicobar Islands	*	*	*	*	*	*
Telangana	4.6	3.3–5.8	43	1.9	1.0-2.7	17
**India**	**8.1**	**7.8–8.4**	**2,469**	**5.1**	**4.8–5.3**	**1543**

*Insufficient sample size

### Multivariable logistic regression

Multivariable binary logistic regression results for undiagnosed depression on CIDI-SFscale by selected characteristics are shown in Table 3. The results show that older adults belonging to urban areas (Odds ratio, OR: 0.80; 95% CI: 0.67–0.96) were less likely than their rural counterparts to suffer from undiagnosed depression. Female older adults were more likely (OR: 1.49; 95% CI: 1.24–1.79) to be undiagnosed for depression. Older adults not covered under any health insurance scheme, having any other physical or mental impairment except depression, having family history of Alzheimer’s/Parkinson’s disease/Psychotic disorder, who rated poor on self-rated health and life satisfaction were more likely than their counterparts of going undiagnosed for probable major depression. Association of selected characteristics with undiagnosed depression measured on the combined scale are also shown in Table 3. All the associations, except covered under any health insurance, remained unchanged when measured on combined scale.

**Table 3:** Multivariable logistic regression estimates for undiagnosed depression cases among older adults, India, 2017-18

Background characteristics	Adjusted Odds Ratio of Undiagnosed Depression on	
	CIDI-SF Scale	Combined Scale
**Place of residence**		
Rural	Ref.	Ref.
Urban	0.80* [0.67–0.96]	0.80* [0.65–0.98]
**Sex**		
Male	Ref.	Ref.
Female	1.49*** [1.24–1.79]	1.66*** [1.30–2.12]
**Marital status**		
Currently married	Ref.	Ref.
Widowed	1.63 [0.76–3.48]	2.02 [0.73–5.58]
Divorced/Separated/Deserted/Others	0.95 [0.40–2.26]	1.30 [0.42–4.04]
**Living arrangement**		
Living alone	Ref.	Ref.
Living with spouse and/or others	1.13 [0.51–2.47]	1.39 [0.49-4.0]
Living with spouse and children	1.06 [0.48–2.32]	1.36 [0.48–3.90]
Living with children and others	0.73* [0.55–0.97]	0.79 [0.57–1.09]
Living with others	0.92 [0.64–1.33]	1.05 [0.68–1.62]
**Religion**		
Hindu	Ref.	Ref.
Muslim	1.05 [0.84–1.32]	1.02 [0.77–1.37]
Others	1.18 [0.90–1.57]	0.90 [0.61–1.32]
**Caste/tribe**		
Scheduled caste	Ref.	Ref.
Scheduled tribe	0.47*** [0.35–0.63]	0.50*** [0.35–0.71]
Other backward classes	1.11 [0.92–1.34]	1.02 [0.80–1.32]
None of the above	0.97 [0.79–1.2]	0.85 [0.66–1.12]
**Education**		
No schooling	Ref.	Ref.
Less than 5 years completed	1.33* [1.03–1.73]	1.36 [0.93–1.97]
5–9 years completed	1.07 [0.88–1.29]	1.03 [0.79–1.33]
10 or more years completed	0.91 [0.71–1.16]	0.96 [0.70–1.33]
**Work status**		
Currently working	Ref.	Ref.
Worked in past but currently not working	1.01 [0.85–1.20]	1.13 [0.90–1.42]
Never worked	0.62*** [0.49–0.77]	0.60*** [0.46–0.80]
**MPCE quintile**		
Poorest	Ref.	Ref.
Poorer	0.89 [0.73–1.08]	0.69* [0.54–0.88]
Middle	1.02 [0.81–1.30]	0.93 [0.67–1.28]
Richer	1.12 [0.90–1.38]	0.99 [0.75–1.29]
Richest	1.40** [1.13–1.72]	1.26 [0.96–1.65]
**Region**		
North	Ref.	Ref.
Central	2.21*** [1.77–2.76]	2.47*** [1.84–3.32]
East	1.07 [0.81–1.30]	0.90 [0.68–1.18]
North-East	0.89 [0.65–1.23]	0.72 [0.45–1.13]
West	1.25 [0.98–1.60]	1.62** [1.17–2.23]
South	0.50* [0.39–0.64]	0.57** [0.41–0.79]
**Health insurance coverage**		
No	Ref.	Ref.
Yes	0.79* [0.65–0.95]	0.79 [0.61–1.04]
**Any other diagnosed neurological/psychiatric issues except depression**		
No	Ref.	Ref.
Yes	0.99 [0.70–1.39]	1.28 [0.88–1.86]
**Any physical/mental impairment**		
No	Ref.	Ref.
Yes	1.96*** [1.61–2.39]	1.92*** [1.49–2.48]
**Family history of Alzheimer’s/Parkinson’s disease/Psychotic disorder**		
No	Ref.	Ref.
Yes	1.67*** [1.33–2.10]	2.00*** [1.52–2.63]
**Self-rated health**		
Good	Ref.	Ref.
Moderate	1.64*** [1.34–2.01]	1.79*** [1.32–2.43]
Poor	3.60*** [2.90–4.47]	4.22*** [3.01–5.84]
**Life satisfaction**		
Low	Ref.	Ref.
Medium	0.58*** [0.48–0.69]	0.52*** [0.40–0.66]
High	0.47*** [0.40–0.56]	0.35*** [0.27–0.44]
Constant	0.04*** [0.02–0.09]	0.02*** [0.01–0.06]
Number of observations	30,637	30,392
Degree of freedom	35	35
Chi-square	703.5***	580.1***
***p < 0.001, **p < 0.01, *p < 0.05		

## Discussion and conclusion

This study has examined the burden of undiagnosed depression among older adults in India using a large-scale population representative household survey. We used CIDI-SF and a combined scale (CIDI-SF and CES-D) to identify undiagnosed depression among older adults. 5% of older adults were undiagnosed for depression on the combined scale. When converted into numbers, 6.8 million older adults in India were undiagnosed for depression on the combined scale for the year 2019. These statistics indicate that undiagnosed depression among the older adults is an important public health challenge that India is facing.

The burden of undiagnosed depression is likely to grow in the country due to the ongoing population ageing in the country. Even after being common than dementia, geriatric depression is not given ample attention as it goes heavily undiagnosed and untreated [26]. Multiple studies have established the significant impact of late life depression on the quality of life [27, 28].Female older adults are observed to be more likely than the male older adults to bear the burden of undiagnosed depression on both the CIDI-SF scale and the combined scale. This finding is in line with the previous research that indicates that female older adults are less likely to seek treatment for ailments compared to their male counterparts [29–31]. In addition, evidence also suggests discrimination against female older adults in health care expenditure and distressed health care financing [32, 33]. Our findings call for greater focus on screening for depression among the female older adults in India.

There is a clear rural urban differential in undiagnosed geriatric depression on both the CIDI-SF and the combined scale. Our study results show a lesser chance of being undiagnosed for depression among the urban older adults in comparison to their rural counterparts. Multiple studies from India and abroad show similar results on rural-urban differentials of geriatric depression [34–36]. A major reason for the same could be the low treatment seeking behaviour among rural population owing to the lack of facilities and institutional support.

Living arrangement is an important factor while understanding depression among older adults. Though our model does not produce significant results for other living arrangement patters, those older adults who are living with their children and others are observed to have lesser odds of not diagnosing for depression rather than living alone.

On both CIDI-SF and the combined scale of measurement, older adults not covered under any health insurance scheme had higher vulnerability towards being undiagnosed for depression. Literature establishes that the lack of insurance often acts as a hindrance for undergoing screening and receiving treatment for depression [37–40]. As per LASI Wave-1 report, 26% of the households in the country are covered under any health insurance scheme. When narrowed down, only 18.1% of the older adults were covered under the Publicly funded health insurance schemes (PFHI) [41]. Ensuring the coverage of schemes among the target population is as important, if not more, as introducing the schemes.

The presence of co-morbid disorders, such as other physical or mental impairment, having family history of Alzheimer’s/Parkinson’s disease/Psychotic disorder, etc., also put older adults at higher risk of undiagnosed depression. Studies have found that the presence of such disorders makes the detection of conditions like depression difficult, as the symptoms might be confused with the ones that are associated with the co-morbidities [32]. It could also be the case that when multiple conditions are present, the undivided attention is given to the physical ailments and the mental health issues are side-lined. In this regard, one should also read that self-rated health levels are found to be a strong predictor of undiagnosed depression among older adults. Those who reported having lower levels of self-rated health are at higher odds of not getting diagnosed for depression. Simultaneously, those who have reported a higher life-satisfaction have lower levels of undiagnosed depression. As in the case of presence of other morbidities, older adults who rate their health levels might be considering only their physical ailments and functional disabilities, making the cognitive impairments go unnoticed.

These findings call for holistic screening of older adults who visit health facilities with associated co-morbidities or physical impairments.

Our study is not devoid of limitations. The question on self-reporting of diagnosed depression is subject to the risk of recall bias among the respondents. Secondly, though the CIDI-SF and CES-D scales are widely used for screening of depression, the use of the same by field interviewers may not strictly qualify as clinical diagnosis of depression. Third, the cross-sectional nature of the LASI wave 1 does not allow to establish causal linkages. Fourth, our analyses could not explore the reasons for undiagnosed depression among the study population as the relevant information is not available in LASI wave 1. Nevertheless, it is one of the first ever attempts to measure the burden of undiagnosed depression among older adults residing in a large and diverse country like India. Our findings are likely to provide key inputs for formulating appropriate policies and programmes for the older adults in India in particular and other low- and middle- income countries in general.

In the context of the results, it is important that the policies and programs of the country take into consideration these findings to improve the diagnosis of depression among older adults. Efforts should be made to address both the demand and supply sides of diagnosis and treatment for geriatric depression. Structural interventions, such as improving the knowledge and attitudes of the service providers and older adults, integration of depression screening with physical health check-ups, collaborative care models of coordination of primary health care and mental health services at local levels, etc. would be beneficial for addressing the burden of undiagnosed depression among the older adults. Effective efforts are also required to improve the discussions on mental health and related issues to eliminate the taboo surrounding the same. Given that India’s older adults’ population is likely to reach 324 million (19% of the total population) by 2040^41^, it is important for the country to adopt a targeted approach focusing on early diagnosis and treatment of depression among older adults.

### Electronic supplementary material

Below is the link to the electronic supplementary material.

Supplementary Material 1

## Data Availability

The study uses secondary data which is available in the public repository of International Institute for Population Sciences, Mumbai, accessible through. https://www.iipsindia.ac.in/content/lasi-wave-i.

## Abbreviations

CIDI-SF: Composite International Diagnostic Interview– Short Form
CES-D: Centre for Epidemiological Studies- Depression scale
OR: odds ratio
CI: Confidence interval
DALY: Disability Adjusted Life years
LASI: Longitudinal Ageing Study in India
MPCE: Monthly per capita consumption expenditure
