# Supplementary material for: Burden of undiagnosed depression among older adults in India: a population based study
Source: BMC Psychiatry. 2024 Apr 30;24:330. doi: 10.1186/s12888-024-05684-5 (PMC11061895; doi:10.1186/s12888-024-05684-5)
Supplement: Supplementary file 1 — Supplementary Material 1 [file 12888_2024_5684_MOESM1_ESM.docx]

Supplementary Table 1: Sample characteristics of the study population, India (2017–18)

| **Background characteristics** | **Proportion (%)** | **Number (N)** |
| --- | --- | --- |
| **Place of residence** |  |  |
| Rural | 70.6 | 22,196 |
| Urban | 29.4 | 9,268 |
| **Sex** |  |  |
| Male | 47.4 | 14,931 |
| Female | 52.6 | 16,533 |
| **Marital status** |  |  |
| Currently married | 61.6 | 19,391 |
| Widowed | 36.2 | 11,389 |
| Divorced/Separated/Deserted/Others | 2.2 | 684 |
| **Living arrangement** |  |  |
| Living alone | 5.7 | 1,787 |
| Living with spouse and/or others | 20.3 | 6,397 |
| Living with spouse and children | 40.6 | 12,779 |
| Living with children and others | 27.6 | 8,696 |
| Living with others | 5.8 | 1,805 |
| **Religion** |  |  |
| Hindu | 82.2 | 25,871 |
| Muslim | 11.3 | 3,548 |
| Others | 6.5 | 2,045 |
| **Caste/tribe** |  |  |
| Scheduled caste | 18.9 | 5,949 |
| Scheduled tribe | 8.1 | 2,556 |
| Other backward classes | 45.2 | 14,231 |
| None of the above | 27.8 | 8,728 |
| **Education** |  |  |
| No schooling | 56.5 | 17,783 |
| Less than 5 years completed | 11.5 | 3,598 |
| 5-9 years completed | 17.8 | 5,611 |
| 10 or more years completed | 14.2 | 4,472 |
| **Work status** |  |  |
| Currently working | 30.8 | 9,679 |
| Worked in past but currently not working | 42.8 | 13,470 |
| Never worked | 26.4 | 8,315 |
| **MPCE quintile** |  |  |
| Poorest | 21.7 | 6,829 |
| Poorer | 21.7 | 6,832 |
| Middle | 20.9 | 6,590 |
| Richer | 19.2 | 6,038 |
| Richest | 16.5 | 5,175 |
| **Region** |  |  |
| North | 12.6 | 3,960 |
| Central | 20.9 | 6,593 |
| East | 23.6 | 7,439 |
| North-East | 3.0 | 935 |
| West | 17.1 | 5365 |
| South | 22.8 | 7,172 |
| **Health insurance coverage** |  |  |
| No | 82.0 | 25,796 |
| Yes | 18.0 | 5,668 |
| **Any other diagnosed neurological/psychiatric issues except depression** |  |  |
| No | 97.5 | 30,663 |
| Yes | 2.5 | 801 |
| **Any physical/mental impairment** |  |  |
| No | 90.8 | 28,565 |
| Yes | 9.2 | 2,899 |
| **Family history of Alzheimer’s/Parkinson’s disease/Psychotic disorder** |  |  |
| No | 95.0 | 29,887 |
| Yes | 5.0 | 1,577 |
| **Self-rated health** |  |  |
| Good | 29.9 | 9,398 |
| Moderate | 46.5 | 14,650 |
| Poor | 23.6 | 7,416 |
| **Life satisfaction** |  |  |
| Low | 30.8 | 9,705 |
| Medium | 23.5 | 8,024 |
| High | 43.7 | 13,735 |
| **Total** |  | 31,464 |

Supplementary Table 2: Number and percentage of older adults age 60+ by measured and diagnosed depression status, India (2017–18)

**ST 2A: Depressed on CIDI-SF Vs depressed on CES-D**

|  | **CIDI-SF Depression: Yes** | **CIDI-SF Depression: No** | **Total** |
| --- | --- | --- | --- |
| **CES-D Depression: Yes** | 1,579 | 7,595 | 9,174 |
| **CES-D Depression: No** | 0 | 21,218 | 21,218 |
| **Total** | 1,579 | 28,813 | 30,392 |

**ST 2B: Depressed on CIDI-SF Vs diagnosed (self-reported) depression**

|  | **CIDI-SF Depression: Yes** | **CIDI-SF Depression: No** | **Total** |
| --- | --- | --- | --- |
| **Diagnosed Depression: Yes** | 36 | 194 | 230 |
| **Diagnosed Depression: No** | 1,543 | 28,619 | 30,162 |
| **Total** | 1,579 | 28,813 | 30,392 |

**ST 2C: Depressed on CES-D Vs diagnosed (self-reported) depression**

|  | **CES-D Depression: Yes** | **CES-D Depression: No** | **Total** |
| --- | --- | --- | --- |
| **Diagnosed Depression: Yes** | 147 | 83 | 230 |
| **Diagnosed Depression: No** | 9,036 | 21,151 | 30,187 |
| **Total** | 9,183 | 21,234 | 30,417 |
